# PAIN CATASTROPHIZING, KINESIOPHOBIA, AND EXERCISE ADHERENCE IN POSTOPERATIVE CARDIOVASCULAR SURGERY PATIENTS: THE MEDIATING ROLE OF EXERCISE SELF-EFFICACY

**DOI:** 10.2340/jrm.v57.43853

**Published:** 2025-11-25

**Authors:** Jingfang WANG, Yufang CHEN

**Affiliations:** Department of Cardiovascular Surgery, Pingjiang General Hospital, the First Affiliated Hospital of Soochow University, Suzhou, China

**Keywords:** pain catastrophizing, self-efficacy, kinesiophobia, exercise adherence, postoperative cardiovascular surgery

## Abstract

**Objective:**

To explore the mediating role of exercise self-efficacy in the relationship between pain catastrophizing, kinesiophobia, and exercise adherence in patients post-cardiovascular surgery.

**Design:**

Cross-sectional study.

**Subjects/Patients:**

A total of 278 adult patients who underwent elective cardiovascular surgeries between March 2023 and February 2025 at a tertiary hospital in China.

**Methods:**

Participants completed standardized instruments measuring pain catastrophizing, kinesiophobia, exercise self-efficacy, and exercise adherence. Statistical analysis was performed using SPSS 29.0, R 4.4.5, and Zstats 1.0, including mediation analysis.

**Results:**

Pain catastrophizing significantly negatively correlated with exercise self-efficacy (*r* = –0.830, *p*<0.001) and adherence (*r* = –0.953, *p* < 0.001), while positively correlating with kinesiophobia (*r* = 0.295, *p* < 0.001). Kinesiophobia negatively correlated with self-efficacy (*r* = –0.252, *p* < 0.001) and adherence (*r* = –0.277, *p*<0.001). Exercise self-efficacy positively correlated with adherence (*r* = 0.699, *p*<0.001). Mediation analysis revealed that self-efficacy partially mediated the relationship between pain catastrophizing and adherence (β = –1.11, *p*<0.001) and between kinesiophobia and adherence (β = –0.30, *p* < 0.001).

**Conclusion:**

Exercise self-efficacy is a critical mediator in how psychological factors like pain catastrophizing and kinesiophobia influence exercise adherence post-cardiovascular surgery. Enhancing self-efficacy may improve rehabilitation outcomes.

## LAY ABSTRACT

After heart surgery, it is important for patients to follow an exercise programme to help them recover. However, many patients struggle to stick with these exercises. This study looks at how certain mental and emotional factors, such as fear of pain and worrying too much about discomfort, can make it harder for people to stay active after surgery. We found that if patients feel more confident about their ability to exercise, they are more likely to stick with their rehabilitation programme, even if they worry about pain or fear injury. This research helps us understand how to better support heart surgery patients by addressing their fears and boosting their confidence, making it easier for them to recover fully and improve their quality of life.

Cardiovascular surgery (CVS) constitutes the primary therapeutic modality for a spectrum of cardiac pathologies, having been extensively refined through clinical practice and supported by robust evidence of therapeutic efficacy (1). These interventions encompass a diverse array of techniques, including but not limited to coronary artery bypass grafting (CABG), ligation of patent ductus arteriosus, cardiac valve annuloplasty, and heart valve replacement (HVR) (2). Cardiovascular surgery, as an invasive therapeutic modality, frequently necessitates postoperative immobilization due to factors such as indwelling catheter management and sedation-analgesia protocols. Prolonged bed rest compromises patients’ mobility and self-care capacity, precipitating muscle mass reduction and subsequent development of postoperative kinesiophobia (3). Early ambulation mitigates symptoms of postoperative complications, improves patient functional capacity, and enhances continuity of rehabilitation outcomes and quality of life (QoL) (4). Although postoperative exercise plays a vital role in improving recovery for patients after cardiovascular surgery, information from the Agency for Healthcare Research and Quality (AHRQ) shows that merely around 20% of these patients participate in cardiac rehabilitation programmes. Furthermore, adherence rates to these programmes are significantly lower among women, ethnic minorities, and individuals from low-income socioeconomic backgrounds (5). Suboptimal adherence predisposes patients to prolonged functional recovery and heightened risks of secondary complications including thromboembolic events, musculoskeletal deconditioning, and cardiovascular event recurrence. Systematic examination of modifiable biopsychosocial determinants influencing rehabilitation compliance – particularly psychological barriers (e.g., kinesiophobia), self-efficacy deficits, and environmental constraints – enables healthcare providers to develop targeted behavioural interventions (6). Emerging evidence underscores the multilevel determinants of rehabilitation adherence, with socioeconomic determinants (particularly health literacy disparities mediated by educational attainment and financial constraints impacting resource access) and clinical complexity factors (types of CVS and pain perception after surgery) demonstrating significant associations with exercise regimen persistence (7). Socioeconomic determinants further reveal that patients with stronger family/friend support systems demonstrate enhanced time allocation for exercise regimens and sustained motivation to maintain physical activity (8, 9). In addition, growing evidence indicates that various psychological elements, such as high levels of pain catastrophizing and kinesiophobia, correlate with poor adherence to postoperative exercise programmes. Conversely, exercise self-efficacy has a beneficial effect on rehabilitation compliance after cardiovascular surgery (10). A study from China revealed that patients with acute coronary syndrome (ACS) exhibit elevated levels of kinesiophobia, and pain catastrophizing and psychogenic anxiety were identified as serial mediators in the pathway linking pain perception to heightened kinesiophobia (11). These findings underscore the necessity for healthcare professionals to systematically monitor patients’ cognitive and psychological adaptations during rehabilitation. Targeted interventions aimed at mitigating maladaptive pain appraisals (e.g., cognitive restructuring of catastrophic thinking patterns) and addressing anxiety rooted in pathological illness perceptions may disrupt this deleterious mediation cascade, thereby reducing exercise-related fear behaviours.

Despite an increasing acknowledgement of psychosocial aspects in postoperative recovery, there has been minimal research dedicated to exploring the interconnections between pain catastrophizing, kinesiophobia, exercise self-efficacy, and exercise adherence among patients after cardiovascular surgery (post-CVS patients). Up to now, only a handful of studies have thoroughly analysed the concurrent relationships among these 4 factors within this specific surgical demographic. Understanding the mediating mechanisms through which cognitive and emotional factors (especially pain catastrophizing and exercise self-efficacy) influence patterns of behavioural adherence is crucial for developing tailored nursing interventions. Fig. 1 presents the proposed mediation model.

**Fig. 1:**
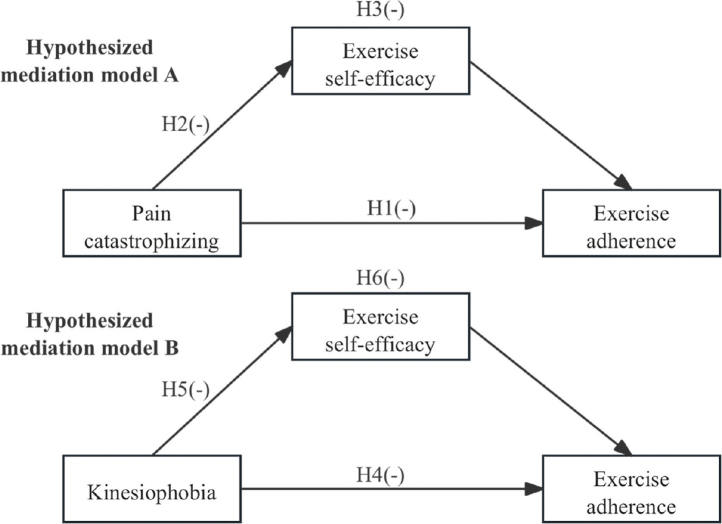
The hypothesized mediation models. (–) Negative predictive effect.

Drawing on the Fear-Avoidance Model and Self-Efficacy Theory (12), we put forward the following hypotheses:

(H1) Pain catastrophizing is a negative predictor of exercise adherence.

(H2) Pain catastrophizing has an unfavourable effect on exercise self-efficacy.

(H3) The relationship between pain catastrophizing and exercise adherence is mediated by exercise self-efficacy.

(H4) Kinesiophobia negatively predicts exercise adherence.

(H5) Kinesiophobia negatively influences exercise self-efficacy.

(H6) Exercise self-efficacy serves as a mediator in the correlation between kinesiophobia and exercise adherence.

## METHODS

### Study design and setting

This cross-sectional research took place from March 2023 to February 2025 at the Department of Cardiac Macrovascular Surgery in the First Affiliated Hospital of Soochow University, located in Suzhou City, Jiangsu Province, China. The investigation follows the STROBE guidelines for reporting observational studies.

### Participants

In this study, post-CVS patients were recruited by convenience sampling. Patients were included if: (a) age 18–80 years old; (b) the type of surgery received includes but is not limited to extracorporeal or non-corporeal coronary artery bypass grafting, pacemaker implantation, arterial catheterization ligation, mitral valve and/or tricuspid valve replacement and aortic valve replacement; (c) the patient is conscious after the operation and has normal visual, auditory, and verbal functions and clear verbal expression; (d) there are no contraindications to cardiac rehabilitation as specified in China Cardiac Rehabilitation and Secondary Prevention (13): these include uncontrolled unstable angina pectoris, uncontrolled severe arrhythmias, and uncontrolled hypertension, defined as a resting systolic blood pressure exceeding 180 mmHg or a resting diastolic blood pressure greater than 100 mmHg; (e) patients who give informed consent and voluntarily cooperate with the investigation. The patients were excluded if: (a) those with Alzheimer’s disease, depression, anxiety, or other disorders that interfere with data collection or who are unable to communicate properly; (b) those with comorbid other serious organic diseases or hearing impairment; (c) patients already enrolled in other studies of psychological interventions or post-cardiovascular interventions.

The determination of the sample size for this research was carried out using *a priori* power analysis with the assistance of G*Power software (version 3.1; https://www.psychologie.hhu.de/arbeitsgruppen/allgemeine-psychologie-und-arbeitspsychologie/gpower). Key statistical parameters were set, reflecting a significance level (α) of 0.05, a statistical power of 95% (1-β = 0.95), and a medium effect size (f² = 0.02) based on similar clinical studies (14). The initial computation yielded a minimum requirement of 252 participants to detect significant associations. To address potential attrition, an additional 10% buffer was incorporated, culminating in a final recruitment target of 280 participants. In total, 280 individuals were recruited for the survey in this study, although 2 participants were excluded due to their questionnaires being completed in less than 240 s.

### Procedure and quality control

Data were collected via an anonymous web-based questionnaire hosted on Questionnaire Star (Ranxing Information Technology Co., Ltd, Changsha, China) (15). Two nurses with bachelor’s degrees, who had received training, were chosen as researchers before the survey commenced. The questionnaire was administered during the survey when patients were in a stable emotional state. A personal approach was employed for the survey, which involved clarifying the study’s aims to the participants, securing their informed consent, and allowing them to use the QR code to complete the questionnaire. Additionally, the participants were informed about necessary precautions while filling out the questionnaire. The questionnaires were screened at the end of questionnaire collection, excluding questionnaires that took < 240 s to complete, questionnaires with consistent answers, and questionnaires with missing answers. Immediately after each survey, the completeness of the questionnaire and whether there were obvious logical errors were checked, and the errors or missing contents were corrected/added after questioning.

### Measurements

*Demographic and clinical characteristics.* A questionnaire created by the researchers collected general information encompassing sex, age, marital status, educational attainment, employment status, religious affiliations, annual per capita household income (in CNY), medical expenses, challenges associated with various types of cardiovascular diseases (CVS), and the classification of cardiac function as per the ACC/AHA Clinical Practice Guideline Recommendation Classification System (16).

*Pain catastrophizing.* The Chinese Vision Pain Catastrophizing Scale (PCS-CV) employed in this research was originally developed by Yap et al. (17). This self-report instrument comprises 13 items across 3 dimensions: rumination (items 8, 9, 10, 11), exaggeration (items 6, 7, 13), and helplessness (items 1, 2, 4, 5, 12). Respondents rate each item using a 5-point Likert scale, yielding total scores that range from 0 to 52, where higher scores indicate greater levels of pain catastrophizing. The total scale demonstrated a Cronbach’s alpha of 0.95, while the alpha coefficients for the individual dimensions were as follows: 0.92 for rumination, 0.86 for exaggeration, and 0.91 for helplessness, indicating robust reliability and validity.

*Kinesiophobia.* The study utilized the Tampa Scale for Kinesiophobia Heart of Chinese Vision (TSK-CV Heart) as described by Lei et al. (18). This instrument is composed of f4dimensions comprising 17 items. These dimensions encompass perceived cardiac danger (items 3, 8, 11, and 16), fear of injury (items 1, 7, 9, and 13), exercise avoidance (items 2, 4, 12, 14, and 17), and diminished self-functioning (items 5, 6, 10, and 15). Evaluation was conducted using a 4-point Likert scale, where responses range from 1 (strongly disagree) to 4 (strongly agree). Items 4, 8, 12, and 16 utilized reverse scoring. The total possible score spans from 17 to 68; higher scores indicate an increased level of fear regarding exercise. The Cronbach’s alpha for the entire scale is 0.859, while the coefficients for the individual dimensions range from 0.743 to 0.821, demonstrating sound reliability and validity.

*Exercise self-efficacy.* In this research, we utilized the Self-Efficacy for Exercise Scale (SEE) (19), which was adapted for the Chinese context by Lee et al. (20) in the same year. This scale includes 9 items, each rated on a scale from 0 to 10, where a score of 0 reflects a lack of confidence and a score of 10 represents a high level of confidence. The overall score is calculated by averaging the scores of all items, with a higher total indicating a greater sense of self-efficacy related to exercise. The reliability and validity of this scale are affirmed by a Cronbach’s alpha coefficient of 0.931.

*Exercise adherence.* The Exercise Rehabilitation Adherence Scale was utilized for this investigation (21). This scale was originally developed for heart failure patients undergoing home-based cardiac rehabilitation, but we consider it appropriate for our study population of post-cardiovascular surgery patients for several reasons. First, major cardiovascular surgery is often accompanied by a decline in cardiac function, similar to that experienced by heart failure patients. Second, the scale items, which assess prescription compliance and monitoring compliance, are relevant to the exercise rehabilitation context and psychological measurement characteristics of our population. The scale comprises 2 dimensions and 11 items, with the first 5 items pertaining to the dimension of “prescription compliance” and the last 6 items relating to “monitoring compliance”. The dimension of “prescription compliance” includes items 1–5, while the “monitoring compliance” dimension consists of items 6–11. Each item is evaluated using a 5-point Likert scale, with response options ranging from never to always, assigned scores from 1 to 5, respectively. A higher score indicates increased adherence to exercise rehabilitation among patients. The overall Cronbach’s alpha for the scale was found to be 0.905, with a coefficient of 0.895 for the “prescription compliance” dimension and a coefficient of 0.910 for the “monitoring compliance” dimension. Additionally, the total folded reliability was calculated at 0.724, with the re-test reliability also standing at 0.724 and a separate re-test reliability assessed at 0.599. The total fold-half reliability measured 0.724, while the re-test reliability reached 0.902, demonstrating satisfactory reliability and validity.

### Data analysis

Data analysis and processing were conducted using R (version 4.4.5; R Foundation for Statistical Computing, Vienna, Austria), along with Zstats v1.0 (available at www.zstats.net) and SPSS 29.0 (IBM Corp, Armonk, NY, USA). Continuous variables were represented as mean ± standard deviation (M ± SD). The normality of data was assessed through Shapiro–Wilk tests (α = 0.05) and normal P–P plots. We defined a statistically significant difference as *p* ≤ 0.05. Comparative analyses for the data in Table I were performed using a *t*-test and the F-test. For correlation analysis, Pearson correlation (for normally distributed data) and Spearman correlation (for non-normally distributed data) were employed. The mediation model, with exercise adherence as the dependent variable, was developed and evaluated using the bootstrap method with 5,000 resampled iterations to assess the mediation effect. If the 95% confidence interval of the mediation effect did not encompass 0, then the mediation effect was deemed significant, forming a theoretical model of interrelationships. A 2-sided test was applied at the α = 0.05 level, and a difference was considered statistically significant when *p* < 0.05.

**Table I:** Sociodemographic and clinical characteristics (*n* = 278)

Category	*n*	%	t/F	*p*-value
Gender				
Male	137	49.28	1.12	0.26
Female	141	50.72		
Age				
< 50 years	132	47.5	1.73	< 0.01
≥ 50 years	146	52.5		
Marital status				
Married=0	74	26.6	0.34	0.79
Unmarried=1	70	25.2		
Divorced=2	69	24.8		
Widowed=3	65	23.4		
Occupational status				
Currently employed	100	36.0	0.41	0.67
Unemployed	100	36.0		
Retirement	78	28.1		
Medical payment				
Agricultural insurance	85	30.6	0.19	0.82
Urban medical insurance	117	42.1		
Self-financed	76	27.3		
Education				
Junior high school and below	70	25.2	0.31	0.82
High school	73	26.3		
Associate degree	62	22.3		
Bachelor’s degree and above	73	26.3		
Annual household income per capita (CNY)				
< 10,000	93	33.5	1.59	0.21
10,000–100,000	86	30.9		
> 100,000	99	35.6		
Religious belief				
Non-religious	141	50.7	–1.26	0.21
Religious	137	49.3		
Difficulty of types of CVS				
Grade 1	198	71.2	52.85	< 0.01
Grade 2	22	7.9		
Grade 3	26	9.4		
Grade 4	21	7.6		
Grade 5	9	3.2		
Grade 6	2	0.7		
Cardiac function class (NYHA)				
II	195	70.1	1.73	< 0.01
III	45	16.2		
IV	38	13.7		

Difficulty of types of CVS, in order of difficulty from easy to difficult: Grade 1: arterial duct ligation. Grade 2: atrial tumour resection, atrial and ventricular defect repair, partial myocardial resection. Grade 3: valve replacement, discoplasty. Grade 4: coronary artery bypass grafting. Grade 5: Bentall’s procedure, Wheat’s procedure, David’s procedure, Act III corrective procedure. Grade 6: aortic (Compound A procedure for coarctation of the aorta; Act IV procedure). NYHA: Class I: No limitation of physical activity; ordinary physical activity does not cause undue fatigue, palpitation, or dyspnoea. Class II: Slight limitation of physical activity; comfortable at rest but ordinary physical activity results in fatigue, palpitation, or dyspnoea. Class III: Marked limitation of physical activity; comfortable at rest but less than ordinary activity causes fatigue, palpitation, or dyspnoea. Class IV: Unable to carry out any physical activity without discomfort; symptoms of cardiac insufficiency at rest.

## RESULTS

### Demographic and clinical characteristics of the samples

The study sample comprised 278 patients who underwent post-CVS procedures, including 137 males (49.28%) and 141 females (50.72%), with an average age of 50.72 ± 12.3 years. The sociodemographic analysis indicated a relatively even distribution among age categories (< 50 years: 47.5%; ≥ 50 years: 52.5%) and marital statuses (married: 26.6%; unmarried: 25.2%; divorced: 24.8%; widowed: 23.4%). Clinically, a significant portion of the participants (71.2%) experienced Grade 1 difficulty during cardiovascular surgery, while 70.1% were categorized as New York Heart Association (NYHA) Class II in terms of cardiac function. Detailed sociodemographic and clinical information can be found in Table I. Furthermore, variations in age (*t* = 1.173, *p* < 0.01), types of surgical difficulties associated with CVS (*F* = 52.85, *p* < 0.01), and cardiac functional classification (*F* = 1.73, *p* <0.01) exhibited a significant correlation with pain catastrophizing. No significant associations were observed between pain catastrophizing and religious beliefs (*p* > 0.05).

### Descriptive statistics and correlation among pain catastrophizing, kinesiophobia, exercise self-efficacy, and exercise adherence

The analysis of correlations indicated significant links between pain catastrophizing, kinesiophobia, exercise self-efficacy, and exercise adherence among patients following cardiovascular surgery (Table II). A marked negative correlation was found between pain catastrophizing and exercise self-efficacy (*r* = –0.830, 95% CI: –0.863 to –0.789, *p* < 0.001), as well as with exercise adherence (*r* = –0.953, 95% CI: –0.963 to –0.941, *p* < 0.001). Conversely, a positive correlation emerged between pain catastrophizing and kinesiophobia (*r* = 0.295, 95% CI: 0.184 to 0.399, *p* < 0.001). Kinesiophobia also exhibited strong negative correlations with both exercise self-efficacy (*r* = –0.252, 95% CI: –0.359 to –0.139, *p* < 0.001) and exercise adherence (*r* = –0.277, 95% CI: –0.383 to –0.165, *p* < 0.001). Furthermore, exercise self-efficacy displayed a strong positive correlation with exercise adherence (*r* = 0.754, 95% CI: 0.698 to 0.800, *p* < 0.001). It was also found that exercise self-efficacy served as a partial mediator in the connection between pain catastrophizing and exercise adherence (*r* = –0.754, 95% CI: –0.649 to –0.578, *p* < 0.001).

**Table II:** Descriptive statistics and correlation among pain catastrophizing, kinesiophobia, exercise self-efficacy, and exercise adherence

Variables	M ± SD	Pain catastrophizing	Kinesiophobia	Exercise self-efficacy	Exercise adherence
Pain catastrophizing	23.19 ± 13.99	1	/	/	/
Kinesiophobia	33.31 ± 13.99	0.295 (0.184~0.399)***	1	/	/
Exercise self-efficacy	7.14 ± 2.71	–0.830 (–0.863~–0.789)***	–0.252 (–0.359~–0.139)***	1	/
Exercise adherence	29.89 ± 14.82	–0.953 (–0.963~–0.941)***	–0.277 (–0.383~–0.165)***	0.754 (0.698–0.800)***	1

*** *p*<0.001.

### Analysis of mediating effects among pain catastrophi-zing, kinesiophobia, exercise self-efficacy, and exercise adherence

**Fig. 2:**
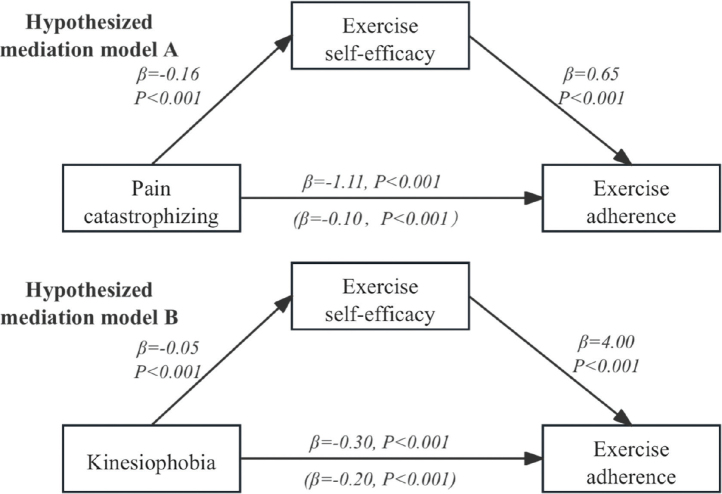
The hypothesized mediation models.

Fig. 2A illustrates that the standardized coefficient for pain catastrophizing in relation to exercise adherence dropped from –0.10 to –1.11 (95% CI: –1.18 to –1.05, *p* < 0.001), demonstrating a strong statistical significance after incorporating the mediator of exercise self-efficacy into the model while controlling for covariates. This finding indicates that exercise self-efficacy serves a partial mediating role between pain catastrophizing and exercise adherence, with the mediating effect representing 8.26% of the overall effect. Additionally, Fig. 2B shows that the standardized coefficient for kinesiophobia affecting exercise adherence fell from –0.20 to –0.30 (95% CI: –0.38 to –0.10, *p* < 0.001), which was also statistically significant, with the mediating effect constituting 66.33% of the total effect. Further information can be found in Tables III and IV.

**Table III:** Regression analysis among pain catastrophizing, kinesiophobia, exercise self-efficacy, and exercise adherence

Model	Paths	β	SE	*p*-value	95% CI
A	Pain catastrophizing to exercise self-efficacy	–0.16	0.01	< 0.001	–0.17~–0.15
	Pain catastrophizing to exercise adherence	–1.11	0.03	< 0.001	–1.18~–1.05
	Exercise self-efficacy to exercise adherence	0.65	0.18	< 0.001	0.31~0.99
B	Kinesiophobia to exercise self-efficacy	–0.05	0.01	< 0.001	–0.07~–0.03
	Kinesiophobia to exercise adherence	–0.30	0.14	< 0.001	–0.38~–0.10
	Exercise self-efficacy to exercise adherence	4.00	0.22	< 0.001	3.56~4.43

**Table IV:** Analysis of mediating effects among pain catastrophizing, kinesiophobia, exercise self-efficacy and exercise adherence

Model	Effect	Estimate	Boot SE	95% CI	Mediation
A	Indirect	–0.10***	0.028	–0.16~–0.05	8.26%
	Direct	–1.11***	0.033	–1.18~–1.05	91.74%
	Total	–1.21***	0.097	–1.35~–0.97	100.00%
B	Indirect	–0.20***	0.048	–0.30~–0.11	66.33%
	Direct	–0.10*	0.296	–0.18~–0.02	33.67%
	Total	–0.29***	0.064	–0.42~–0.17	100.00%

* *p* = 0.018,

*** *p* < 0.001.

## DISCUSSION

### Correlation of demographic and clinical characteristics with pain catastrophizing

The results of this study show that patients aged ≥ 50 years demonstrated significantly higher pain catastrophizing scores compared with those < 50 years (*t* = 1.73, *p* < 0.01). The positive association between advanced age (≥ 50 years) and pain catastrophizing aligns with prior studies suggesting that older adults may experience heightened pain-related anxiety due to physiological decline, prolonged recovery, and reduced functional resilience (22). Notably, age-related declines in endogenous pain modulation and prefrontal cortical control over catastrophizing cognitions (e.g., rumination) may exacerbate maladaptive pain appraisals (23). Patients with poorer cardiac function (class III/IV) exhibited higher pain catastrophizing (*F* = 1.73, *p* < 0.01), likely due to activity limitations, recurrent symptoms (e.g., dyspnoea), and fear of exacerbating cardiac strain during exercise (24). This aligns with evidence that reduced exercise tolerance in heart failure patients amplifies kinesiophobia and catastrophizing cognitions (25). Higher CVS grades were linked to elevated catastrophizing scores, possibly reflecting the psychological toll of invasive procedures, prolonged ICU stays, and uncertainty regarding recovery (26). Such findings underscore the need for preoperative psychological screening in high-risk surgical cohorts. No significant associations were observed between pain catastrophizing and gender, marital status, education, income, or religious beliefs (*p* > 0.05).

### Mediation analysis of pain catastrophizing, kinesiophobia, exercise self-efficacy, and exercise adherence

The current mediation analysis revealed that exercise self-efficacy served as a partial mediator in the connection between pain catastrophizing and exercise adherence (β = –1.11, *p* < 0.001, 95% CI = –1.18 to –1.05), as well as in the relationship between kinesiophobia and exercise adherence (β = –0.30, *p* < 0.001, 95% CI = –0.38 to –0.10). This indicates that higher levels of exercise self-efficacy reduce the adverse effects of pain catastrophizing and kinesiophobia on adherence, which is consistent with the findings of Zhou et al. regarding patients experiencing post-total knee arthroplasty pain, pain catastrophizing, and kinesiophobia (27). Pain catastrophizing, characterized by maladaptive cognitive-emotional responses to pain (e.g., magnification, rumination, helplessness), may lead to activity avoidance due to exaggerated threat appraisals, thereby reducing adherence and hindering functional recovery after CVS. These results corroborate Bandura’s self-efficacy theory, wherein negative cognitive appraisals diminish confidence in one’s physical capabilities, resulting in reduced engagement in rehabilitation activities (28). Notably, 71.2% of patients had grade 1 surgical difficulty, and 70.1% were classified as NYHA class II, indicating that even those with relatively mild physiological impairments may experience significant psychological barriers, necessitating early psychosocial interventions (29). Targeted strategies to enhance self-efficacy – such as graded exercise exposure (e.g., progressive task mastery) and mastery experiences (e.g., structured goal-setting with incremental challenges) – can buffer the detrimental effects of pain catastrophizing on adherence. Clinically, integrating cognitive-behavioural techniques (e.g., cognitive restructuring to address catastrophic thinking, behavioural activation to reinforce adaptive coping) may improve postoperative outcomes by fostering confidence in physical abilities and reducing fear-avoidance behaviours (30). Future interventions should address socioeconomic disparities in postoperative care while employing multimodal approaches (e.g., self-monitoring tools, vicarious learning via peer modelling) to optimize self-efficacy and long-term recovery trajectories (31).

### Limitations

Three methodological constraints warrant consideration when interpreting these findings. The predominant reliance on non-probabilistic sampling from a single tertiary cardiovascular centre introduces potential selection bias, thereby constraining the generalizability of observed patterns across diverse healthcare settings and demographic subgroups. It is noteworthy that while AHRQ reports consistently indicate lower adherence rates among female patients in broader clinical populations, our study sample exhibited a balanced gender distribution (male: 49.28%, female: 50.72%). This discrepancy may be attributed to our specific recruitment strategy or regional demographic characteristics, and may not fully represent the gender disparities documented in larger national datasets. Future studies with purposive sampling strategies may further elucidate this phenomenon. Furthermore, the observational nature inherent to cross-sectional designs precludes temporal sequencing analysis, fundamentally limiting causal inference between psychosocial constructs and rehabilitation behaviours. To address these constraints, future investigations should prioritize multicentre prospective cohort studies employing stratified random sampling frameworks, complemented by experimental paradigms such as nurse-led behavioural RCTs.

### Conclusion

This research illustrates that self-efficacy in exercise serves as a unique mediator in the connection between psychological obstacles (such as pain catastrophizing and kinesiophobia) and adherence to exercise in patients who have undergone cardiovascular surgery. The mediation analysis indicated that exercise self-efficacy partially mediated the effects of both pain catastrophizing (accounting for 8.26% of the overall effect) and kinesiophobia (making up 66.33% of the overall effect) on adherence to exercise. Importantly, a higher level of exercise self-efficacy reduced the adverse effects of pain catastrophizing on exercise adherence and acted as a crucial pathway through which kinesiophobia impacted exercise participation. These findings underscore the necessity of integrating self-efficacy enhancement strategies into postoperative rehabilitation programmes, particularly interventions targeting kinesiophobia reduction. Clinically, fostering patients’ confidence in their physical capabilities may significantly mitigate exercise avoidance behaviours and improve long-term recovery outcomes. Future research should validate these mechanisms in diverse populations and explore longitudinal effects of self-efficacy-based interventions.

## References

[1] Shemin RJ. The future of cardiovascular surgery. Circulation 2016; 133: 2712–2715. 10.1161/CIRCULATIONAHA.116.023545 27324365

[2] Jiang J-H, Liu N, Yang Y-L. Observation on the effect of early goal-oriented activities on the application of rehabilitation for patients after cardiac major vascular surgery. J Community Med 2023; 17: 910–914. 10.19790/j.cnki.JCM.2023.17.09

[3] Zhang C, Li X, Li H, Xie M. Abrupt formation of intracardiac thrombus during patent foramen ovale closure by three-dimensional transesophageal echocardiography. Echocardiography 2022; 39: 643–644. 10.1111/echo.15326 35238060

[4] Raidou V, Mitete K, Kourek C, Stamatopoulos S, Karmpaliotis D, El-Sherif N, et al. Quality of life and functional capacity in patients after cardiac surgery intensive care unit. World J Cardiol 2024; 16: 436–447. 10.4330/wjc.v16.i8.436 39221189 PMC11362807

[5] Rubin R. Although cardiac rehab saves lives, few eligible patients take part. JAMA 2019; 322: 386–388. 10.1001/jama.2019.8604 31314061

[6] Sanches EE, Aupers E, Sakran N, Navalta J, Kostka T, Pouwels S. Barriers and facilitators in rehabilitation in chronic diseases and after surgery: is it a matter of adherence? Cureus 2021; 13: e20173. 10.7759/cureus.20173 35003999 PMC8723784

[7] Evie Herlinda, Wiyadi, Purwanto E. Relationship between socio demographics and compliance with taking hypertension medication in the elderly. Formosa J Appl Sci 2023; 2: 805–822. 10.55927/fjas.v2i5.3995

[8] Abraham LN, Sibilitz KL, Berg SK, Tang LH, Risom SS, Lindschou J, et al. Exercise-based cardiac rehabilitation for adults after heart valve surgery. Cochrane Database Syst Rev 2021; 5: CD010876. 10.1002/14651858.CD010876.pub3 33962483 PMC8105032

[9] Gray E, Smith C, Bunton R, Skinner M. Perceptions and experiences of engaging in physical activity following coronary artery bypass graft surgery. Physiother Theory Pract 2022; 38: 2841–2855. 10.1080/09593985.2021.1989733 34666600

[10] Shajrawi A, Khalil H, Al-Sutry M, Qader RA, Eid AbuRuz M. Exercise self-efficacy, perceived benefits, and barriers to exercise among patients following acute myocardial infarction. J Cardiovasc Nurs 2021; 36: E11–E19. 10.1097/JCN.0000000000000810 33833189

[11] Wang YY, Zhang J, Sun YJ, Bai XJ, Xu HF. The chain mediation effect of pain catastrophizing and psychogenic anxiety on the relationship between pain and exercise fear in patients with acute coronary syndrome. J Nurs Manag 2024; 2: 103–107. 10.3969/j.issn.1671-315x.2024.02.003

[12] Sandborgh M, Johansson A-C, Söderlund A. The relation between the fear-avoidance model and constructs from the social cognitive theory in acute WAD. Pain Res Manag 2016; 2016: 8281926. 10.1155/2016/8281926 27999473 PMC5141534

[13] Chinese Society of Rehabilitation Medicine, Cardiovascular Disease Committee, Editor-in-Chief. Guidelines for cardiac rehabilitation and secondary prevention in China (2018 edition). Beijing: Peking University Medical Press; 2018.

[14] Faul F, Erdfelder E, Buchner A, Lang AG. Statistical power analyses using G*Power 3.1: tests for correlation and regression analyses. Behav Res Methods 2009; 41: 1149–1160. 10.3758/BRM.41.4.1149 19897823

[15] Questionnaire Star. Questionnaire Star (Online survey platform). Available at: https://www.wjx.cn

[16] Goff DC Jr, Lloyd-Jones DM, Bennett G, Coady S, D’Agostino RB, Gibbons R, et al. 2013 ACC/AHA guideline on the assessment of cardiovascular risk: a report of the American College of Cardiology/American Heart Association Task Force on Practice Guidelines. Circulation 2014; 129: S49–73. 10.1161/01.cir.0000437741.48606.98 24222018

[17] Yap JC, Lau J, Chen PP, Gin T, Wong T, Chan I, et al. Validation of the Chinese Pain Catastrophizing Scale (HK-PCS) in patients with chronic pain. Pain Med 2008; 9: 186–195. 10.1111/j.1526-4637.2007.00307.x 18298701

[18] Lei M, Liu T, Xiong S, Sang M, Kim C. Sinicization and reliability validity test of exercise fear scale for cardiac patients. China Nurs Manag 2019; 11: 1637–1642. 10.3969/j.issn.1672-1756.2019.11.009

[19] Resnick B, Jenkins LS. Testing the reliability and validity of the Self-Efficacy for Exercise scale. Nurs Res 2000; 49: 154–159. 10.1097/00006199-200005000-00007 10882320

[20] Lee LL, Perng SJ, Ho CC, Hsu HM, Lau SC, Arthur A. A preliminary reliability and validity study of the Chinese version of the self-efficacy for exercise scale for older adults. Int J Nurs Stud 2009; 46: 230–238. 10.1016/j.ijnurstu.2008.09.003 18950769

[21] Yang Z, Sun Y, Wang H, Zhang C, Wang A. A scale for measuring home-based cardiac rehabilitation exercise adherence: a development and validation study. BMC Nurs 2023; 22: 259. 10.1186/s12912-023-01426-2 37550733 PMC10405489

[22] Li CF, He L, Huang YL, Liu Y, Gao MM, Yan JW, et al. Investigation of pain catastrophizing and influencing factors in total knee arthroplasty patients. J Nurs 2020; 35: 22–38. 10.3870/j.issn.1001-4152.2020.23.022

[23] Petrini L, Arendt-Nielsen L. Pain catastrophizing in the elderly: an experimental pain study. Scand J Pain 2024; 24: 20230035. 10.1515/sjpain-2023-0035 38452201

[24] Westerdahl E, Bergh C, Urell C. Patient-reported physical activity, pain, and fear of movement after cardiac surgery: a descriptive cross-sectional study. Scand Cardiovasc J 2024; 58:2393311. 10.1080/14017431.2024.2393311 39158171

[25] Yifan T, Yanling H, Weiyun W, Xiaolin H, Zejuan G, Rong W, et al. Mediation analysis of activities of daily living and kinesiophobia in association between cardiac function and health status of patients with chronic heart failure. Clin Cardiol 2023; 46: 1049–1058. 10.1002/clc.24147 37706605 PMC10540005

[26] Zeng Z, Shen Y, Wan L, Yang X, Hu Q, Luo H, et al. Kinesiophobia in patients after cardiac surgery: a scoping review. BMC Cardiovasc Disord 2024; 24: 469. 10.1186/s12872-024-04140-2 39223455 PMC11370225

[27] Zhou Y, Gao W, Gao S, Guo X, Liu M, Cao C. Pain catastrophizing, kinesiophobia, and exercise adherence in patients after total knee arthroplasty: the mediating role of exercise self-efficacy. J Pain Res 2023; 16: 3993–4004. 10.2147/JPR.S432106 38026453 PMC10676101

[28] Bandura A. Self-efficacy: toward a unifying theory of behavioral change. Psychol Rev 1977; 84: 191-215. 10.1037//0033-295x.84.2.191 847061

[29] Everett B, Salamonson Y, Davidson PM. Bandura’s exercise self-efficacy scale: validation in an Australian cardiac rehabilitation setting. Int J Nurs Stud 2009; 46: 824–829. 10.1016/j.ijnurstu.2009.01.016 19261281

[30] Dibben G, Faulkner J, Oldridge N, Rees K, Thompson DR, Zwisler A-D, et al. Exercise-based cardiac rehabilitation for coronary heart disease. Cochrane Database Syst Rev 2021; 11: CD001800. 10.1002/14651858.CD001800.pub4 34741536 PMC8571912

[31] Zhang R, Zhu C, Chen S, Tian F, Chen Y. Exercise-based cardiac rehabilitation for patients after heart valve surgery: a systematic review and re-evaluation with evidence mapping study. Clin Cardiol 2025; 48: e70117. 10.1002/clc.70117 40130747 PMC11934209

